# FGF19/FGFR4 signaling axis confines and switches the role of melatonin in head and neck cancer metastasis

**DOI:** 10.1186/s13046-021-01888-9

**Published:** 2021-03-10

**Authors:** Liwei Lang, Yuanping Xiong, Nestor Prieto-Dominguez, Reid Loveless, Caleb Jensen, Chloe Shay, Yong Teng

**Affiliations:** 1 Department of Oral Biology and Diagnostic Sciences, Dental College of Georgia, Augusta University, 1120 15th Street, Augusta, GA 30912 USA; 2 Present address: Department of Otolaryngology Head and Neck Surgery, First Affiliated Hospital of Nanchang University, Nanchang, Jiangxi China; 3 Department of Pediatrics, Emory Children’s Center, Emory University, Atlanta, GA USA; 4 Georgia Cancer Center, Department of Biochemistry and Molecular Biology, Medical College of Georgia, Augusta University, Augusta, GA USA; 5 Department of Medical Laboratory, Imaging and Radiologic Sciences, College of Allied Health, Augusta University, Augusta, GA USA

**Keywords:** FGF19/FGFR4 axis, Melatonin, ER stress, Metastases, H3B-6527, HNSCC

## Abstract

**Background:**

There is no consensus about the effective dosages of melatonin in cancer management, thus, it is imperative to fully understand the dose-dependent responsiveness of cancer cells to melatonin and the underlying mechanisms.

**Methods:**

Head and neck squamous cell carcinoma (HNSCC) cells with or without melatonin treatment were used as a research platform. Gene depletion was achieved by short hairpin RNA, small interfering RNA, and CRISPR/Cas9. Molecular changes and regulations were assessed by Western blotting, quantitative RT-PCR (qRT-PCR), immunohistochemistry, and chromatin Immunoprecipitation coupled with qPCR (ChIP-qPCR). The therapeutic efficacy of FGF19/FGFR4 inhibition in melatonin-mediated tumor growth and metastasis was evaluated in orthotopic tongue tumor mice.

**Results:**

The effect of melatonin on controlling cell motility and metastasis varies in HNSCC cells, which is dose-dependent. Mechanistically, high-dose melatonin facilitates the upregulation of FGF19 expression through activating endoplasmic stress (ER)-associated protein kinase RNA-like endoplasmic reticulum kinase (PERK)-Eukaryotic initiation factor 2 alpha (eIF2α)-activating transcription factor 4 (ATF4) pathway, which in turn promotes FGFR4-Vimentin invasive signaling and attenuates the role of melatonin in repressing metastasis. Intriguingly, following long-term exposure to high-dose melatonin, epithelial HNSCC cells revert the process towards mesenchymal transition and turn more aggressive, which is enabled by FGF19/FGFR4 upregulation and alleviated by genetic depletion of the FGF19 and FGFR4 genes or the treatment of FGFR4 inhibitor H3B-6527.

**Conclusions:**

Our study gains novel mechanistic insights into melatonin-mediated modulation of FGF19/FGFR4 signaling in HNSCC, demonstrating that activating this molecular node confines the role of melatonin in suppressing metastasis and even triggers the switch of its function from anti-metastasis to metastasis promotion. The blockade of FGF19/FGFR4 signaling would have great potential in improving the efficacy of melatonin supplements in cancer treatment.

**Supplementary Information:**

The online version contains supplementary material available at 10.1186/s13046-021-01888-9.

## Background

Fibroblast growth factors (FGFs) are a family of 22 different proteins that bind and stimulate a specific group of tyrosine kinase receptors called FGFRs. FGF19 is a non-canonical FGF that can control a broad spectrum of physiological and pathological responses by signaling through its receptor FGFR4 [1–3]. FGF19 was first characterized in hepatocellular carcinoma (HCC) as an oncogenic driver [4, 5]. Previous studies in our laboratory have revealed that FGF19 directly activates FGFR4-GSK3β signaling cascade in HCC cells to promote the epithelial-mesenchymal transition (EMT) by upregulating β-catenin pathway and provide a cytoprotective role against endoplasmic reticulum (ER) stress by promoting Nrf2 pathway [6, 7]. We further demonstrated that inhibition of FGF19 not only suppresses HCC metastasis but also increases its sensitivity to sorafenib by enhancing reactive oxygen species (ROS)-associated apoptosis [8].

Melatonin (MT) is a methoxyindole synthesized and released by the pineal gland and other extrapineal sources, including the oral cavity and the salivary glands [9–11]. There is highly credible evidence outlining the relevance of MT to human physiology and pathology. Although MT principally functions to control sleep-wake, circadian, and seasoning cycles, it also carries out anti-inflammatory, antioxidant, and immunomodulatory actions in different human tissues [9, 10, 12–16]. As to cancer, MT exhibits oncostatic features mitigating cancer at the initiation, progression, and metastasis phases through receptor-dependent and/or -independent mechanisms [13–15, 17–20]. Moreover, induction of apoptosis and endoplasmic reticulum (ER) stress contribute to the beneficial effects of MT in cancer treatment. There are five groups of molecules interfering with ER stress, including drugs directly interacting with the ER signaling, chemical chaperones, protein degradation inhibitors, drugs affecting calcium signaling, and antioxidants [21, 22]. One of the mechanisms of the action of MT is that it functions as a potent antioxidant and free radical scavenger exhibiting sensitizing effects on ER stress-induced apoptosis in cancer cells [21]. Importantly, MT has shown to be an endogenously generated agent helpful in cancer prevention and treatment due to its low toxicity [15, 16]. A good example is that MT can be used as a potential adjuvant treatment in oral cancer therapy [11]. Although most experts advise to avoid extremely high dosages of MT, there is no consensus about its effective dose in cancer management [23, 24]. In order for the better use of MT in the clinic, there is an urgent need for fully understanding the dose-dependent responsiveness of cancer cells to MT and the underlying mechanisms.

Little progress has been made in the treatment of head and neck squamous cell carcinoma (HNSCC) in decades due to the lack of impactful targeted therapies [25, 26]. We have demonstrated that blockade of tumor-derived FGF19/FGFR4 signaling represents a promoting therapeutic strategy to preferentially target FGF19-driven HNSCC [25]. Here, we report for the first time that MT has the ability to induce ER stress-associated FGF19 upregulation in a dose-dependent fashion, which in turn activates FGFR4-Vimentin invasive signaling in HNSCC cells. We also unveil the previously unrecognized influence of FGF19/FGFR4 signaling on the role of MT in metastasis. Results obtained demonstrate the impact of MT dosages on HNSCC malignancy, suggesting inhibition of FGF19-FGFR signaling axis to be a potential strategy for improving the therapeutic efficacy of MT supplements.

## Methods

### Cell lines and culture

HNSCC cell lines HN6, HN12, and HN30 were a gift from Dr. W. Andrew Yeudall and maintained in our lab [25, 27]. Human telomerase-immortalized tonsillar keratinocytes hTERT HAK Clone 41 were a gift from Dr. A. Klingelhutz and Dr. J. Lee (University of Iowa, Iowa City, IA) and cultured in KSFM with 0.2 ng/ml EGF and 30 μg/ml bovine pituitary extract [28]. All cells were used for experiments before passage 10 and cultured in DMEM containing 10% fetal bovine serum at 37 °C in a humidified incubator supplied with 5% CO_2_. Luciferase stable HN30 cells were generated by transduction of pGL4.5 vector (Promega, Madison, WI) and selection of hygromycin for 6 weeks. MT long-term exposure (MT-LE) HN30 cells were generated by exposure to 5 mM MT and maintenance in the medium containing the same dose of MT for more than 10 generations.

### Constructs, reagents, antibodies and standard assays

MT, D-luciferin bioluminescent substrate, alamarBlue Cell Viability Reagent, and Dead Cell Apoptosis Kit were purchased from ThermoFisher Scientific (Waltham, MA). MT stock solution at 1 M was prepared by dissolving it in DMSO in ultrasonic sonication bath. For in vivo assays, MT was dissolved in 20% glycofurol solutions to reduce the potential toxicity to mice. The full-length FGF19 gene was amplified from HN30 cells and cloned into pcDNA3.1 (+) expression vector (Invitrogen, USA) as previously described [7]. FGFR4-speficic inhibitor H3B-6527 was purchased from Selleckchem (Houston, TX). Antibodies that recognize ATF4, PERK, p-PERK, IRE-1, eIF2α, p-eIF2α, E-cadherin, Vimentin, Puma, Noxa, Bcl-2, and Bax were purchased from Cell Signaling Technology (Beverly, MA). Antibodies against FGF19, FGFR4, p-FGFR4 and β-Actin were obtained from Abcam (Cambridge, MA). Western blotting, RT-PCR, wound healing assays, Annexin V FITC/PI double staining, and cell viability assays were carried out as we described previously [1, 7, 25, 27, 29]. Proteins were visualized using a SuperSignal West Pico chemiluminescence kit (Pierce) and quantified with ImageJ Fiji (version 1.2; WS Rasband, National Institute of Health, Bethesda, MD).

### Quantitative RT-PCR (qRT-PCR) and chromatin immunoprecipitation qPCR (ChIP-qPCR)

Total RNA was isolated from cells or tissue using Trizol (Invitrogen, San Diego, CA) according to the manufacturer’s instructions. QRT-PCR analysis was conducted with Applied Biosystems™ Power SYBR™ Green PCR Master Mix (ThermoFisher Scientific) using the StepOne Plus Real Time PCR System (Applied Biosystems, Foster City, CA). Gene expression levels were normalized against β-actin. In this assay, primers for human FGF19 were: 5′-GGAGATCCGCCCAGATGGCTAC-3′ (Forward) and 5′-GGCCTCCAGTCCGGTGACAAGC-3′ (Reverse). Primers for human β-actin were: 5′- GAGCACAGAGCCTCGCCTTT-3′ (Forward) and 5′-TCATCATCCATGGTGAGCTGG-3′ (Reverse). ChIP assay were performed using the anti-ATF4 antibody (Abcam) and the ChIP Assay Kit (Millipore, Burlington, MA) as described previously [7]. Normal rabbit IgG-AC (Santa Cruz Biotechnology, Dallas, TX) was used as a negative control for the antibody. The immunoprecipitated genomic DNA was amplified by qPCR using the PCR primers (Forward: 5′-ACTCCACTTTCCTCGGAATC-3′; Reverse: 5′-TCCCCCTCTTCTTCCACCGC-3′) designed to amplify a proximal promoter region containing the putative ATF4 binding element AARE (5′-CTTGATGCA-3′) on the FGF19 promoter.

### Constructs and gene modifications

The pLKO.1-puro TRC non-targeting short hairpin RNA (shRNA) control (shCTRL) and shRNAs targeting FGF19 (shFGF19–1, shFGF19–2) were obtained from Horizon Discovery (Waterbeach, UK). Lentiviruses were packaged in 293FT cells with an optimized mix of the three packaging plasmids (pLP1, pLP2, and pLP/VSVG) using the ViraPower™ Lentiviral Expression System (Invitrogen, Carlsbad, CA). A small interfering RNA (siRNA) against ATF4 (siATF4) and a non-targeting siRNA (siCTRL) were synthesized by Dharmacon (Lafayette, CO), and transfected into target cells using Lipofectamine RNAiMAX Transfection Reagent (Invitrogen). FGFR4 knockout (KO) HN12 cells were generated using the CRISPR/Cas9 system. Single-guide RNAs (sgRNAs) were designed via an online web tool (http://crispr.mit.edu) to target a region spanning the FGFR4 (transcript variant 1) exon 3 (5′-TGTGCGTCTGTGCTGTGGGC-3′). The synthesized guide sequences were cloned into pSpCas9 (BB)-2A-Puro (PX459) V2.0 (Addgene plasmid # #62988) [30]. After transfection, positive FGFR4 KO clones were screened by western blotting with an antibody against FGFR4 and further verified by sequencing.

### Three-dimensional (3D) invasion assays

The experiment was modified and carried out as we previously described [31, 32]. Briefly, 1 × 10^4^ indicated cells diluted in 50% Matrigel (BD biosciences) were seeded in the center hole of SeedEZ 3D ring (Lena Bioscience, Atlanta, GA). After one-week culture, cells invaded in the SeedEZ™ 3D ring were stained with Texas-red phalloidin (Invitrogen) and imaged under a fluoresce microscope (Zeiss).

### Animal study

Six-week-old NSG mice were purchased from the Jackson Laboratory (Bar Harbor, ME), and all animal experiments were approved by the Institutional Animal Care and Use Committee (IACUC) of Augusta University. An orthotopic tongue tumor model was generated as described previously [26, 33]. For in vivo MT challenge experiment, 2 × 10^5^ HN12 cells were suspended in 50 μl of PBS/Matrigel (3:1) and injected into anterior ~ 1/3 tongue of NSG mice under anesthesia. DMSO or MT (2.5 mM and 5 mM) was intratumorally injected when the tongue xenografts reached ~ 50 mm^3^, and the xenografts were harvested for western blotting and immunohistochemistry (IHC) after 12 h. To assess the effect of FGF19 loss, 2 × 10^4^ luciferase-containing MT-LE and its parental HN30 cells with or without FGF19 knockdown were individually injected into anterior NSG mouse tongue. To determine the efficacy of H3B-6527, NSG mice were randomized to be intratumorally injected with 50 μl sterile saline (vehicle) or H3B-6527 (10 μM) under anesthesia after two-week inoculation with 2 × 10^4^ MT-LE or its parental HN30 cells. The tumor-bearing mice received H3B-6527 treatment every 3 days for 2 weeks. Tumor growth and metastasis were measured externally every week by bioluminescence measurement using a Xenogen IVIS-200 In Vivo Imaging System (PerkinElmer, Waltham, MA). Images were quantified as photons/ second using the Living Image software. The mice were sacrificed at the endpoint, and the primary xenografts and the major organs were then removed for H&E staining and IHC analysis.

### Assessment of FGF19 and MT in HNSCC patients

The present studies in human biological specimens were reviewed and approved by the Institutional Review Board of the First Affiliated Hospital of Nanchang University, China. All samples were obtained with informed consent. The blood samples from thirty-five adult patients who have HNSCC with median age 58 years old were collected from the antecubital vein between 6:00 am and 8:00 am 1 day before surgery. The blood samples in the control group were from ten healthy age-matched people. Within 1 hour after collection, the blood samples were placed immediately on ice until centrifugal sera separation and storage at − 80 °C. The Melatonin ELISA Kit (BioVision, Milpitas, CA) and the Human FGF19 Quantikine® ELISA kit (R&D Systems, Minneapolis, MN) were used for quantitative measurement of MT and FGF19 in blood samples. Tumor tissues from the same cohort of HNSCC patients were collected for IHC to assess FGF19 levels. The clinicopathologic characteristics of sample sets was shown in Supplementary Table S1.

### IHC

IHC was performed as we previously described [25, 33, 34]. Briefly, Paraffin-embedded xenografts or tissues were cut into 3 μm sections and mounted on slides, followed by blocking in 10% of normal goat serum after antigen retrieval in hot citrate buffer. The sections were then incubated with the primary antibodies against FGF19 or Vimentin. Consecutive sections were stained with hematoxylin to help localize cancer tissues and adjacent normal epithelium, and immuno-reactivity was visualized and analyzed using the DAB Kit (Vector Laboratories, Burlingame, CA) under a CCD camera (Olympus, Center Valley, PA). Samples were examined by three investigators who were blind to pathological information. At least ten random microscopic fields were captured per sample, and signal intensity was then semi-quantified using ImageJ Fiji (version 1.2) as described previously [35].

### Statistical analysis

All in vitro experiments were performed in triplicate and statistical analyses were performed by two-tailed Student *t* test using the SPSS software package version 12.0. For in vivo study, animals were randomly chosen, and blinding outcome assessment and concealed allocation were used. In vivo treatment effects were evaluated using a one-way analysis of variance (ANOVA), followed by post-hoc test analysis for individual group comparisons. Results from multiple experiments were expressed as the means ± standard deviations (SDs), and differences were considered statistically significant when *p* < 0.05.

## Results

### MT triggers ER stress-associated FGF19 upregulation in HNSCC cells

**Fig. 1:**
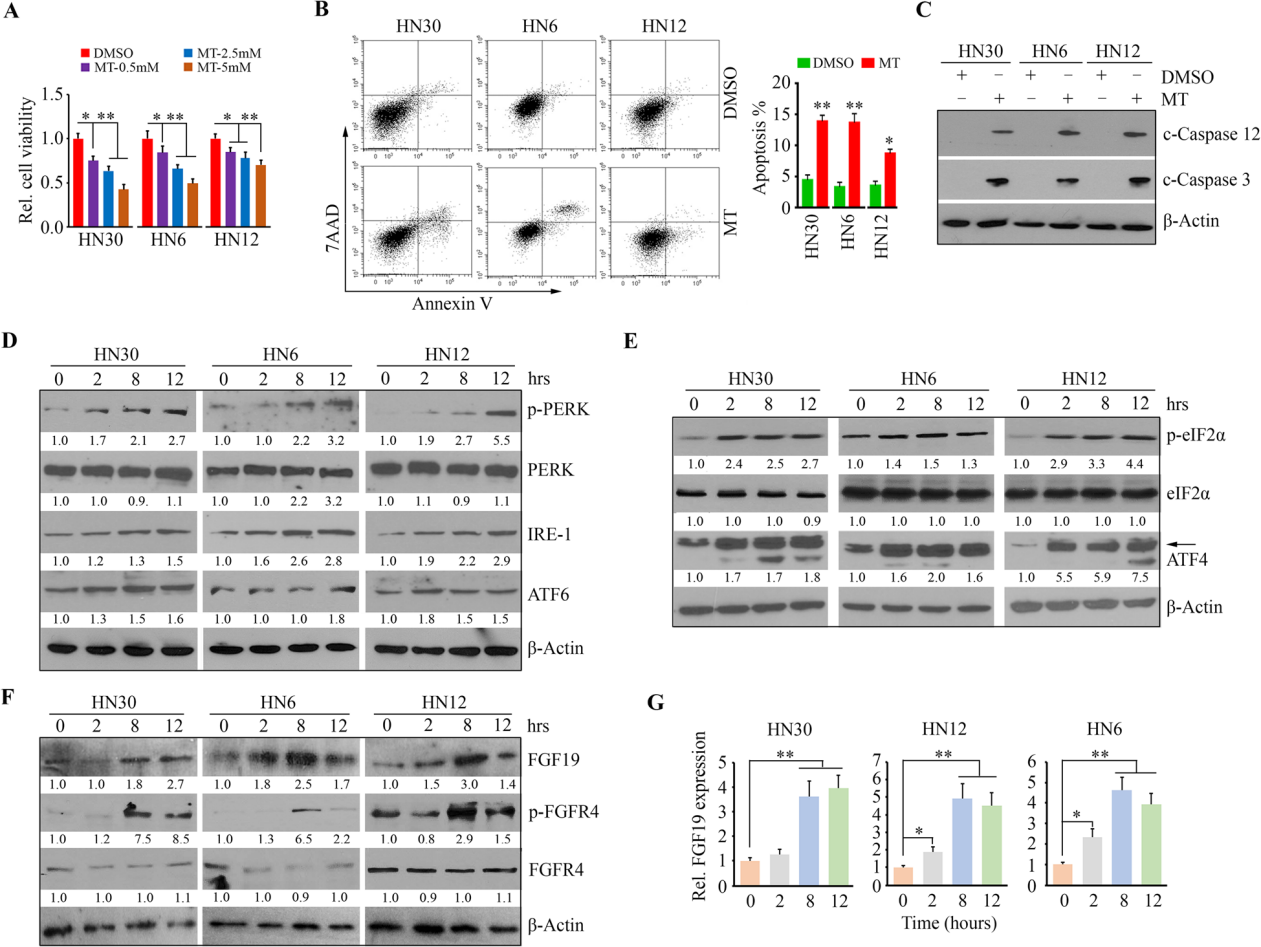
MT induces ER stress-associated upregulation of FGF19 in HNSCC cells. **a** The inhibitory effect of MT on cell viability determined after three-day treatment. **b** The inducible effect of MT on apoptosis determined by Annexin V FITC/PI staining after two-day treatment. Representative results and quantitative data from three independent experiments are shown in the left and right panels, respectively. **c** The effect of MT on the cleavage of Caspase 3 and Caspase 12 determined after two-day treatment. **d** The level changes in ER stress markers within the first 12 h following MT treatment. **e** The activation of elF2α-ATF4 signaling within the first 12 h following MT treatment. **f** The activation of FGF19-FGFR4 axis within the first 12 h following MT treatment. **g** Increased FGF19 mRNA expression within the first 12 h following MT treatment. *p < 0.05; **p < 0.01

We first determined the cytotoxicity of MT in HNSCC cell lines by treating them with varied doses of MT for 3 days. MT exhibited strong effects on cell viability inhibition and apoptosis induction among HNSCC cells examined (Fig. 1a and b), which was consistent with previous studies reporting MT’s anticancer potency [26, 34, 35]. Compared with HN6 and HN30 cells, HN12 cells were more resistant to MT (Fig. 1a and b). An active form of Caspase 3 (c-Caspase 3) was detected in cancer cells treated with MT (Fig. 1c), which confirmed flow cytometry analysis at the molecular level. Interestingly, c-Caspase 12 was also seen in cancer cells following MT exposure (Fig. 1c), suggesting that MT may ignite the apoptotic pathway through ER stress induction. We then treated cells with 5 mM MT following a time course and subjected them to Western blotting with antibodies against ER stress-responsive proteins. Increased protein levels of ATF6 and IRE-1, coupled with enhanced phosphorylation levels of PERK were seen in cells treated with MT within 12 h (Fig. 1d). While the levels of IRE-1 protein and PERK phosphorylation were increased dramatically following MT treatment, ATF6 protein levels were elevated moderately (Fig. 1d). ER stress has been reported to govern cell survival and death through functionally linking to mitochondria [22, 36]. To determine whether MT-induced ER stress-associated death was mediated by the mitochondrial pathway of apoptosis in HNSCC cells, we assessed the changes in mitochondria-localized Bcl-2 family proteins in the presence or absence of MT. Western blotting analysis revealed an upregulation of BH1–3 pro-apoptotic Bcl-2 protein Bax upon MT treatment, which was accompanied by increased BH3-only Bcl-2 proteins Puma and Noxa (Supplementary Figure S1). As BH3-only Bcl-2 family proteins are effectors of canonical mitochondrial apoptosis, which discharge their pro-apoptotic functions through BH1–3 pro-apoptotic proteins, our findings indicate that ER stress triggered by MT induces HNSCC cell apoptosis via mitochondria-dependent pathway.

Increased PERK phosphorylation prompted us to determine eIF2α-activating ATF4 signaling, as it is commonly regulated by PERK when phosphorylated upon ER stress [37]. The levels of phosphorylated eIF2α were markedly elevated at 2 hours after MT treatment, along with the increase of ATF4 (Fig. 1e). ATF4 is a critical regulator involved in ER stress-induced FGF19 upregulation in HCC cells [7]. As ATF4 was elevated in MT treatment, we determined the FGF19 expression following MT treatment in HNSCC cells. MT led to a significant increase in FGF19 protein levels, concomitant with increased phosphorylation levels of its receptor FGFR4 (Fig. 1f). QRT-PCR further showed MT-induced FGF19 upregulation at the mRNA level (Fig. 1g).

### High-dose MT promotes FGF19 upregulation in HNSCC cells via ER stress-induced ATF4 activation

**Fig. 2:**
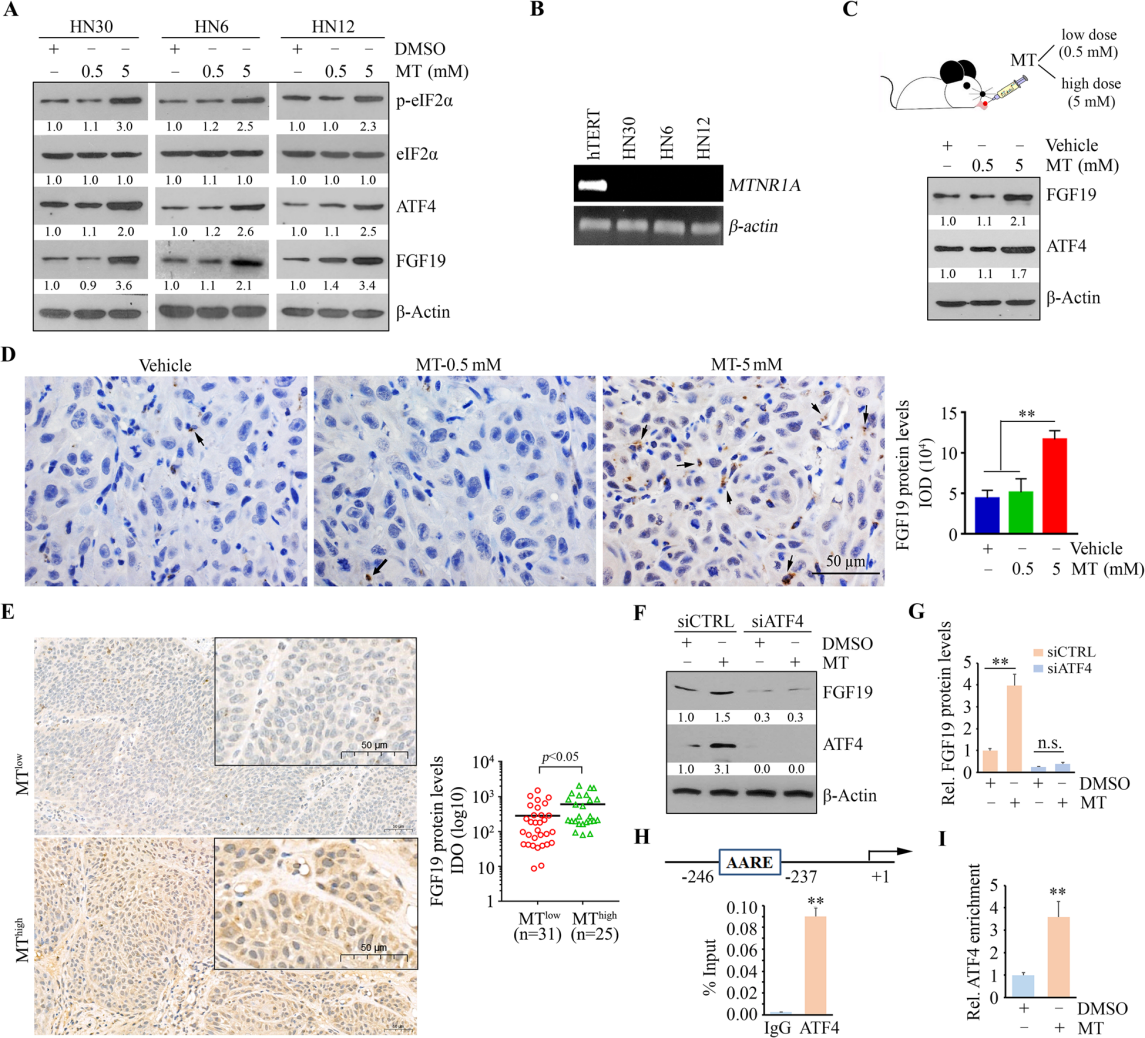
High-dose MT induces upregulation of FGF19 in HNSCC cells via ATF4. **a** The effects of high and low dose of MT on the activation of elF2α-ATF4-FGF19 signaling pathway in the indicated cell lines following MT treatment for 12 h. **b** *MTNR1A* mRNA levels in HNSCC cell lines and normal oral keratinocytes (hTERT). **c**, **d** The effects of high and low dose of MT on the increase of FGF19 levels in the orthotopic mouse tongue tumor model. The xenografts were harvested for western blotting analysis (**c**) after homogenization and IHC with the anti-FGF19 antibody (**d**). In (**d**), arrows indicate FGF19-positive tumor cells in the HN12-derived tumor xenografts. **e** The protein levels of FGF19 in human primary HNSCC tissues with high or low levels of serum MT determined by IHC. In (**d**, **e**), quantitative data are shown in the right panels. **f**, **g** The effect of ATF4 knockdown on FGF19 expression in HN12 cells at either protein (**f**) or mRNA levels (**g**). **h** The binding of ATF4 on the FGF19 gene promoter determined by ChIP-qPCR assay. A putative ATF4 binding site (AARE) on the FGF19 gene promoter is shown in the cartoon (above). **i** The enrichment of ATF4 on the FGF19 gene promoter determined by ChIP-qPCR assay in the presence or absence of 5 mM MT. *p < 0.05; **p < 0.01

Intriguingly, reduction of MT dose from 5 mM to 0.5 mM did not activate eIF2α-ATF4 axis and altered FGF19 protein levels in all three HNSCC cells (Fig. 2a), suggesting that only high-dose MT can induce FGF19 upregulation. To understand whether MT signals through its receptors, we first determined the expression levels of MT receptor 1 A (*MTNR1A*). RT-PCR analysis revealed that normal oral keratinocytes expressed *MTNR1A*, but *MTNR1A* mRNA was not detected in all cancer cell lines examined in our study (Fig. 2b). This observation suggests that MT activates the eIF2α-ATF4-FGF19 pathway through receptor-independent mechanism. We then developed orthotopic NSG mice and intratumorally injected low (0.5 mM) or high dose (5 mM) of MT when HN12-derived xenografts were formed on the mice tongue (Fig. 2c). In accordance with in vitro data, MT at high dose, but not at low dose, enhanced ATF4 protein levels in the isolated xenograft tumors as measured by western blotting and IHC (Fig. 2c and d).

To determine the clinical correlation between serum FGF19 and MT concentrations, we collected blood samples from age-matched healthy people and HNSCC patients. ELISA analysis showed higher levels of serum MT levels, but not serum FGF19 in the blood of HNSCC patients compared with the healthy cohort (Supplementary Figure S2A). Spearman’s rank correlation coefficient analysis demonstrated no good correlation of the serum FGF19 level with MT in the HNSCC patient cohort (Supplementary Figure S2B), suggesting that MT has no significant influence on systemic levels of FGF19 in HNSCC patients. However, IHC data from this cohort rendered that patients with high concentrations of serum MT had significantly higher FGF19 protein levels in tumor tissues than those with low concentrations of serum MT (Fig. 2e). These findings imply that serum MT is one possible contributing factor to the upregulation of FGF19 in HNSCC.

Next, we depleted ATF4 expression in HN12 cells and measured the FGF19 expression in the presence or absence of 5 mM MT (Fig. 2f). While the addition of MT increased FGF19 levels in the knockdown control cells, no changes in FGF19 levels were detected in either MT treated or non-treated cells when ATF4 was depleted (Fig. 2g). ChIP-qPCR assays clearly showed ATF4 bound to the FGF19 promoter in HN12 cells (Fig. 2h) and increased amount of ATF4 at the FGF19 promoter binding site in the presence of 5 mM MT (Fig. 2i), indicating that MT-induced ER stress promotes ATF4 upregulation, which in turn transcriptionally activates FGF19 in HNSCC cells.

### FGF19-FGFR4 signaling axis confines the role of MT in suppressing cell motility via Vimentin upregulation in HNSCC cells

**Fig. 3:**
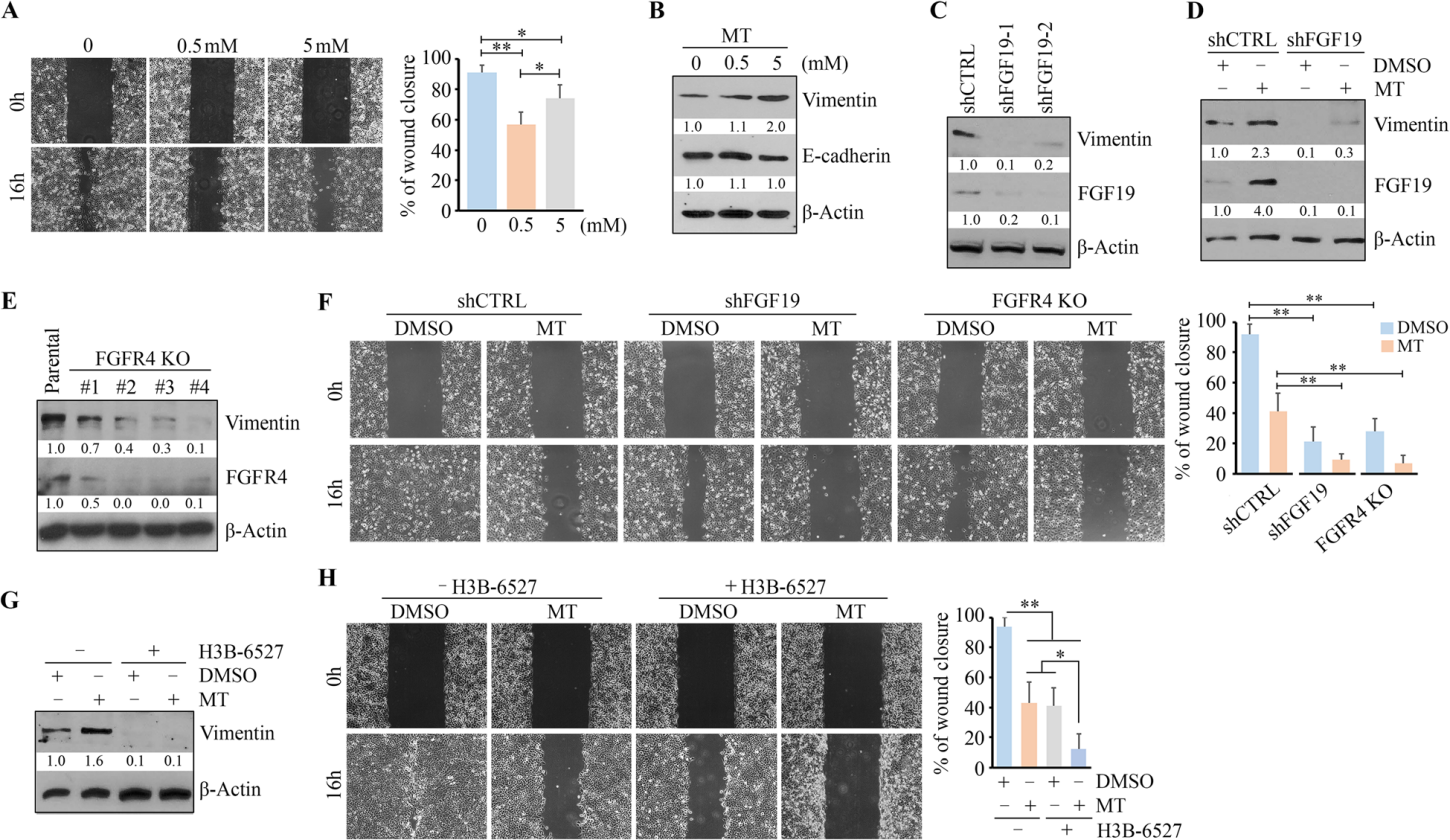
FGF19-FGFR4 signaling axis confines the role of MT in suppressing cell motility via upregulating Vimentin in HNSCC cells. **a** The effect of MT at low and high doses on cell migration determined by wound healing assays within 16 h following treatment. **b** The effect of MT on Vimentin protein levels in HN12 cells determined by Western blotting. **c** The effect of FGF19 knockdown (shFGF19–1 and shFGF19–2) on Vimentin protein levels in HN12 cells determined by Western blotting. **d** The effect of MT on cell migration in HN12 cells on Vimentin protein levels with or without FGF19 knockdown (shFGF19 = shFGF19–1). **e** The effect of FGFR4 KO on Vimentin protein levels in HN12 cells determined by Western blotting. **f** The effect of 5 mM MT on cell migration in HN12 cells with or without the genetic inhibition of FGF19 (shFGF19) or FGFR4 (FGFR4 KO) gene expression. **g**, **h** The effect of MT on Vimentin levels (**g**) and cell migration (**h**) in HN12 cells in the presence or absence of FGFR4 inhibitor H3B-6527. In (**a**, **f**, **h**), representative images of cell migration and quantitative data from three independent experiments are shown in the left and right panels, respectively. *p < 0.05; **p < 0.01

Next, we determined whether MT has an impact on HNSCC cell migration. Wound healing assay showed that while MT repressed wound closure in highly-invasive HN12 cells at 16 h (Fig. 3a), its inhibitory effect was more significant at 0.5 mM compared with 5 mM (Fig. 3a), suggesting that high-dose MT may enhance cell-killing effect but attenuate the role in suppressing cell motility. Intriguingly, elevated Vimentin was seen in cells treated with 5 mM MT, which was accompanied by E-cadherin reduction (Fig. 3b). To examine whether FGF19 was involved in Vimentin upregulation in HNSCC cells, we knocked down the FGF19 gene in HN12 cells. FGF19 knockdown not only repressed Vimentin expression (Fig. 3c) but also abrogated its increase upon high-dose MT exposure and enhanced the inhibitory effect of MT on cell migration (Fig. 3d and f). We then depleted FGFR4 in HN12 cells using the CRISPR/Cas9 system. Three FGFR4 KO clones (#2, #3 and #4) rendered decreased Vimentin protein levels consistently compared with the parental control cells (Fig. 3e). Moreover, knockout of FGFR4 led to a reduction in cell migration and augmented the effect of MT on migration suppression (Fig. 3f). H3B-6527, a highly selective covalent FGFR4 inhibitor, also exhibited a suppressive effect on Vimentin protein levels at 5 μM (Fig. 3g), leading to impaired wound healing capacity in HN12 cells (Fig. 3h). Most importantly, additional H3B-6527 significantly enhanced MT-induced migration inhibition (Fig. 3h), which was consistent with the results from FGF19 or FGFR4 gene depletion. These findings indicate that high-dose MT only has limited effect on suppressing cell motility in HNSCC cells due to the induction of FGF19-FGFR4-Vimentin signaling.

### FGF19-FGFR4 signaling axis switches the role of MT in cell motility when epithelial HNSCC cells were long exposed to high-dose MT

**Fig. 4:**
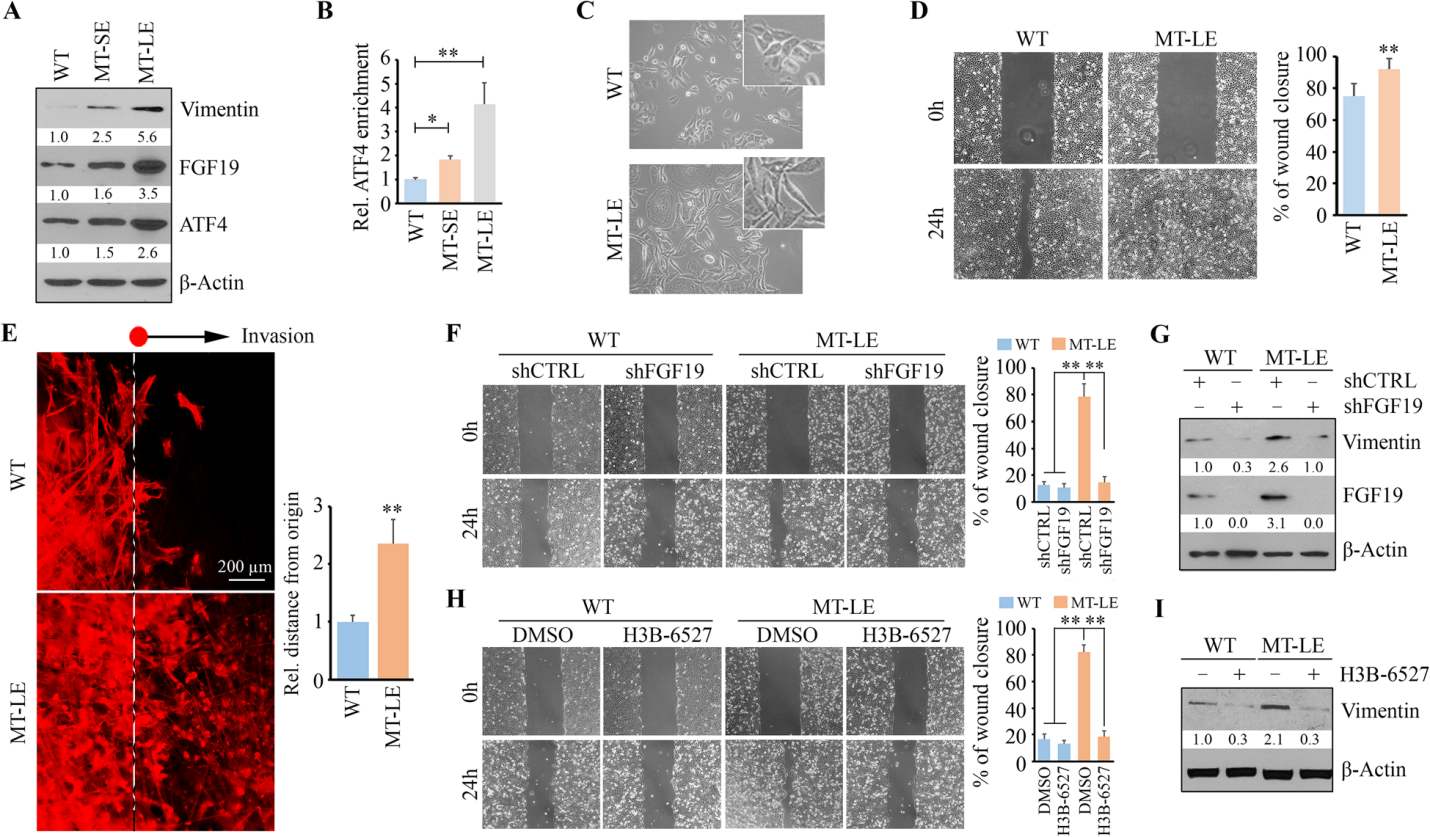
FGF19-FGFR4 signaling axis switches the role of MT in cell motility upon long-term exposure to high-dose MT. **a** Increased levels of ATF4, FGF19, and Vimentin in MT-SE and MT-LE HN30 cells. **b** The increased ATF4 amount on the FGF19 gene promoter in MT-LE HN30 cells than MT-SE HN30 cells determined by ChIP-qPCR. **c** The morphological changes between MT-LE and its parental HN30 cells photographed under an inverted phase-contrast microscopy. **d** Comparison of migration capacity between MT-LE and its parental HN30 cells by wound healing assays. **e** Comparison of ex vivo invasion potential between MT-LE and its parental HN30 cells by SeedEZ™ 3D rings. **f**, **g** The effect of FGF19 knockdown (shFGF19) on cell migration (**f**) and Vimentin protein levels (**g**) in MT-LE HN30 cells. **h**, **i** The effect of H3B-6527 on cell migration (**h**) and Vimentin protein levels (**i**) in MT-LE HN30 cells. In (**d**, **e**, **f**, **h**) representative images and quantitative data from three independent experiments are shown in the left and right panels, respectively. MT-SE, HN30 cells under 12-h exposure of MT; MT-LE, HN30 cells under long-term exposure of MT. *p < 0.05; **p < 0.01

Unlike HN12 cells, HN30 cells are epithelial and noninvasive HNSCC cells. To better understand the role of FGF19 in MT treatment, we used HN30 cells to develop MT-LE cells by maintaining them in medium containing 5 mM MT for more than 10 generations. We first assessed the apoptotic status of MT-LE cells by examining c-Caspase 3 levels. The cleaved Caspase 3 was observed in HN30 cells after short exposure of MT (MT-SE) but it was not detected in MT-LE cells (Supplementary Figure S4), suggesting that HNSCC cells may acquire resistance to MT-induced apoptosis after a long exposure. Strikingly, elevated FGF19 coupling with ATF4 and Vimentin were observed in MT-LE cells compared with its parental cells and MT-SE cells that were only exposed to 5 mM MT for 12 h (Fig. 4a). ChIP-qPCR assays further showed enriched ATF4 bound on the FGF19 promoter in MT-LE cells than that in either its parental or MT-SE cells (Fig. 4b). Morphologically, parental HN30 cells were characterized as “tight colonies” [38] with typical epithelial shape (Fig. 4c). Short-term exposure to MT did not change HN30 morphology (data not shown), but MT-LE cells appeared spindle-shaped and made few contacts with adjacent cells (Fig. 4c), suggesting EMT in HNSCC cells after long-term exposure to high-dose MT. A clear increase in scratch-wound healing capability was noted in MT-LE cells compared with its parental cells (Fig. 4d), which prompted us to further study their invasion abilities in a 3D scaffold. While the parental cells growing in the SeedEZ 3D ring only displayed short-distance invasion, MT-LE cells efficiently invaded at long-distance (Fig. 4e), suggesting that HNSCC cells may gain metastatic potential when long exposed to high-dose MT.

To explore how FGF19 contributes to the switch of MT’s role in cell motility, we overexpressed FGF19 in HN30 cells, which showed increased Vimentin levels along with acquired migration capacity compared with the control cells (Supplementary Figure S3). We then knocked down the FGF19 gene in MT-LE and its parental HN30 cells. HN30 cells showed no effect to FGF19 knockdown on cell migration (Fig. 4f), which may be due to the nature of low FGF19 expression levels. In contrast, FGF19 loss significantly diminished the migration potential of MT-LE cells and was associated with decreased Vimentin (Fig. 4f and g). Consistently, inactivation of FGFR4 by its inhibitor H3B-6527 repressed Vimentin protein expression in MT-LE cells, leading to a reduction in cell migration (Fig. 4h and i).

### Blockade of FGF19/FGFR4 signaling axis attenuates the cervical lymph node metastases (LNMets) of MT-LE HNSCC cells

**Fig. 5:**
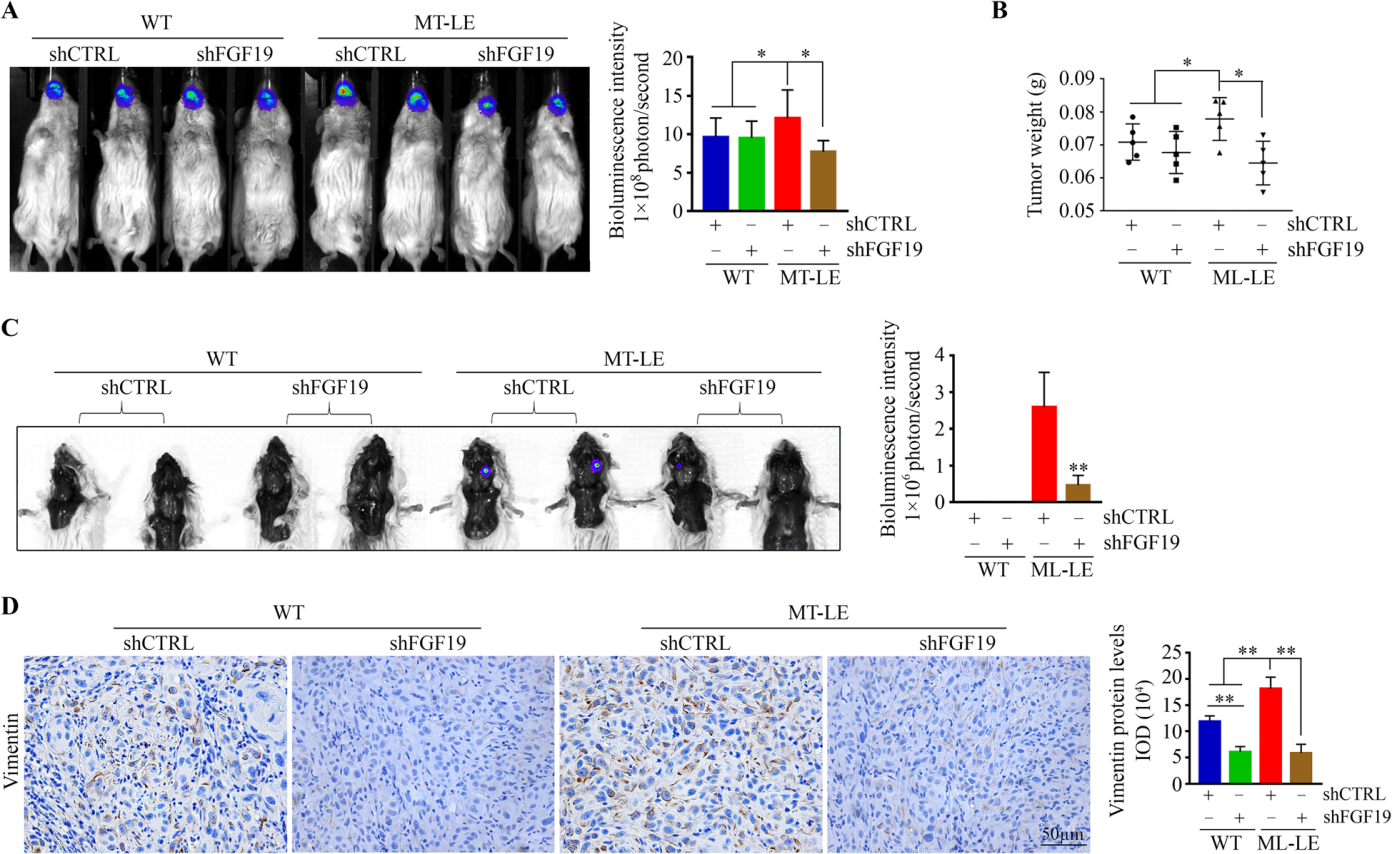
Knockdown of FGF19 attenuates the LNMets of MT-LE HNSCC cells in orthotopic mice of tongue tumors. **a**, **b** Bioluminescence images of tongue tumors (**a**) and tongue tumor weight (**b**) in mice implanted with the indicated cells after a four-week inoculation. **c** Bioluminescence images of tumor dissemination in lymph nodes in mice implanted with the indicated cells after a four-week inoculation. **d** The protein levels of Vimentin in tumor xenografts from mice implanted with the indicated cells determined by IHC. In (**a**, **c**, **d**), representative images and quantitative data are shown in the left and right panels, respectively. *p < 0.05; **p < 0.01

Luciferase-containing MT-LE and its parental HN30 cells with or without FGF19 knockdown were generated and individually injected into the anterior tongue of NSG mice (*n* = 5/group). There were no noticeable changes in tumor growth in mice receiving FGF19 knockdown or its knockdown control cells (Fig. 5a) after a four-week inoculation. A marked increase in tumor growth was observed in mice implanted with knockdown control MT-LE cells (Fig. 5a and b). FGF19 knockdown suppressed xenograft tumor growth derived from knockdown control MT-LE cells (Fig. 5a and b). Moreover, no LNMets were observed in mice that were implanted with knockdown control parental cells after a four-week inoculation, but knockdown control MT-LE cells developed LNMets in mice as illustrated by the bioluminescence signal in mice lymph nodes (Fig. 5c). Most importantly, the bioluminescence was only detected in three out of five mice implanted with FGF19 knockdown MT-LE cells, and the signal intensity was much lower compared with that in mice receiving knockdown control MT-LE cells (Fig. 5c). In addition, higher levels of Vimentin were consistently seen in tumor tissues derived from knockdown control MT-LE cells compared with knockdown control parental cells (Fig. 5d), which were dramatically suppressed in xenograft tumors when FGF19 was depleted (Fig. 5d).

**Fig. 6:**
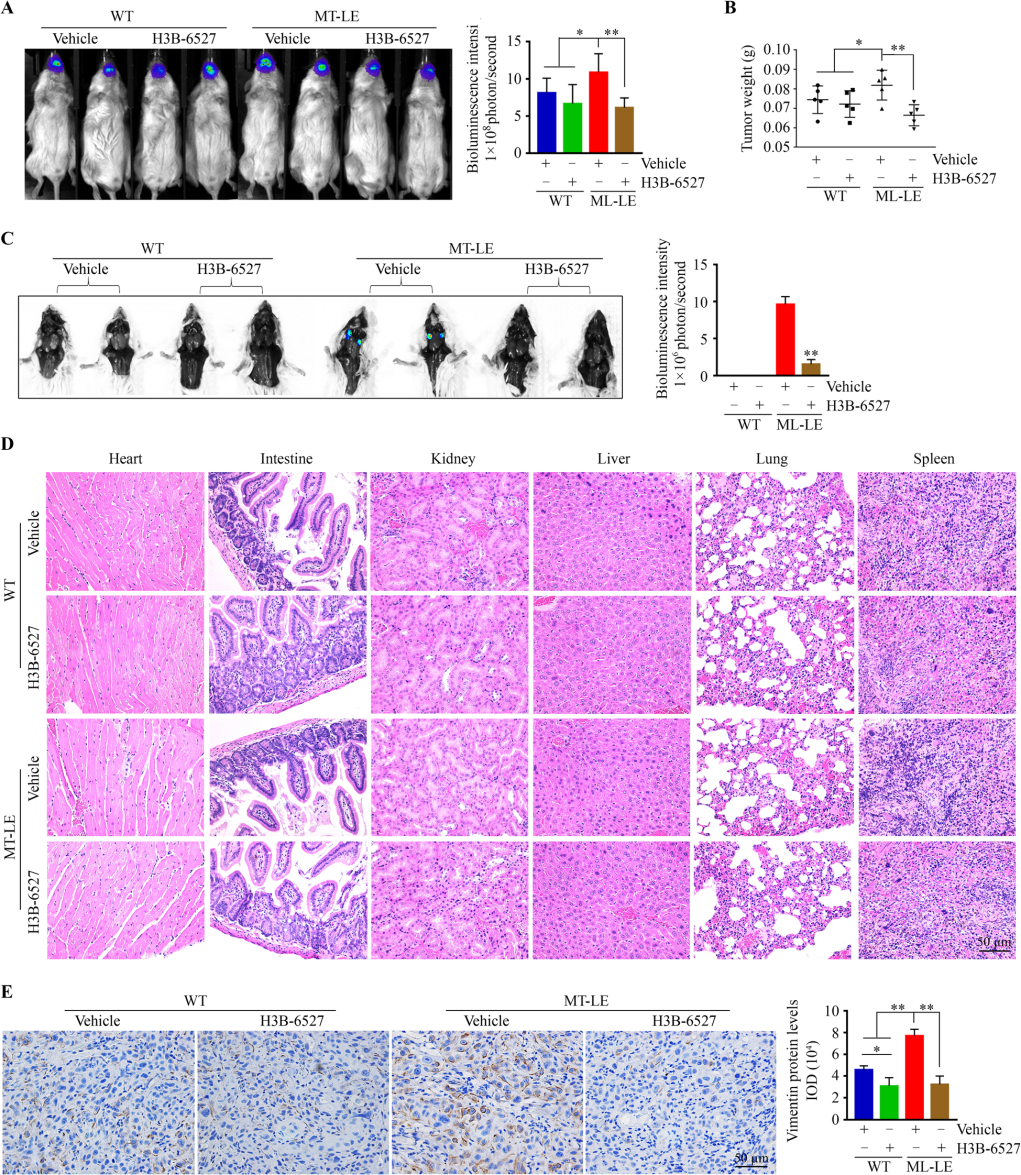
Inactivation of FGFR4 blocks the LNMets of MT-LE HNSCC cells in orthotopic mice of tongue tumors. **a**, **b** Bioluminescence images of tongue tumors (**a**) and tongue tumor weight (**b**) in mice receiving vehicle or H3B-6527 after 2 weeks of intratumoral treatment. **c** Bioluminescence images of tumor dissemination in lymph nodes in mice receiving vehicle or H3B-6527 after a two-week intratumoral treatment. **d** Histology of major organs at the endpoint of each treatment. **e** The protein levels of Vimentin in tumor xenografts from mice treated with or without H3B-6527 determined by IHC. In (**a**, **c**, **d**, **e**), representative images and quantitative data are shown in the left and right panels, respectively. *p < 0.05; **p < 0.01

Tumor burden was also decreased in tumor-bearing mice treated with H3B-6527 (Fig. 6a and b). No tumor cells derived from the parental HN30 cells metastasized into lymph nodes as no bioluminescence was there (Fig. 6c). However, strong bioluminescence signals were detected from lymph nodes in mice that were implanted with MT-LE cells (Fig. 6c), supporting the notion that HNSCC cells become more aggressive when they are exposed to high-dose MT for a long time. After H3B-6527 treatment, only two mice (40%) implanted with MT-LE cells developed tiny secondary tumors in mice lymph nodes at the endpoint (Fig. 6c). H&E staining showed no detectable morphologic changes in the major organs (Fig. 6d) among all four groups, suggesting H3B-6527 has no or litter systemic toxicity. IHC analysis further indicated that increased Vimentin in MT-LE cell-derived tumors was dramatically attenuated by H3B-6527 (Fig. 6e). Taken together, these observations suggest that blockade of FGF19-FGFR4 signaling has the potential to reverse the aggressiveness developed along with long exposure of high-dose MT.

## Discussion

**Fig. 7:**
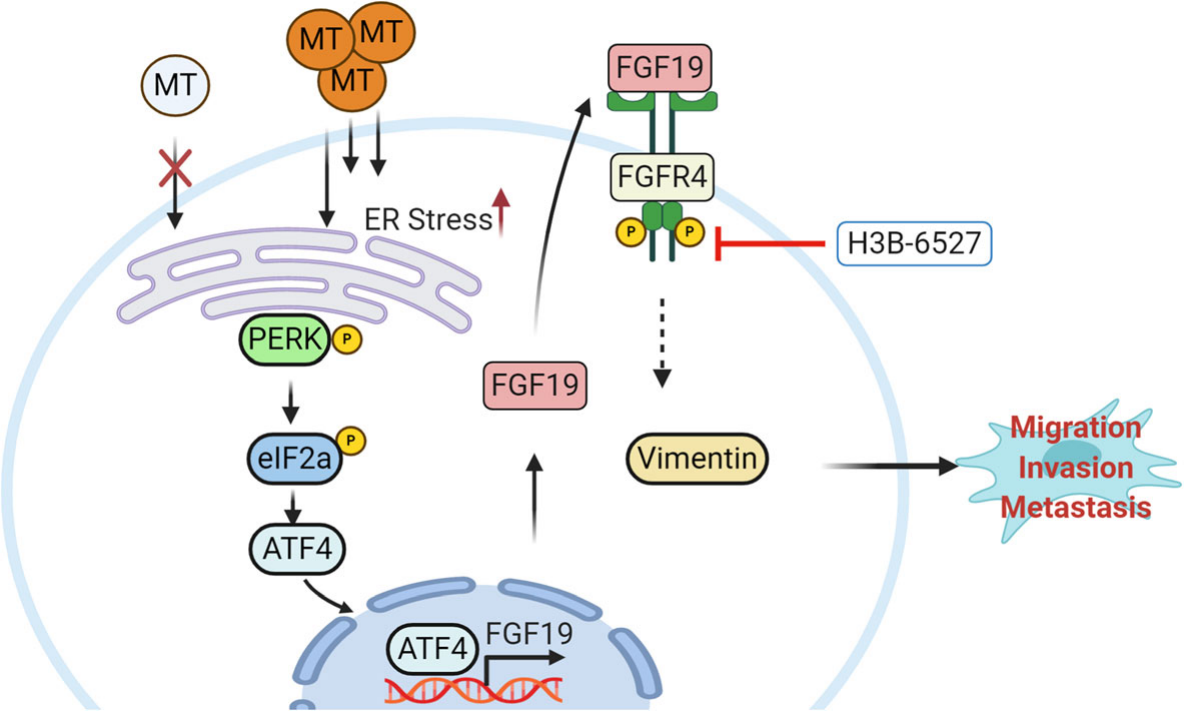
Schematic representation of MT-mediated FGF19 signaling in HNSCC cells. High-dose MT has the potential to upregulate FGF19 gene expression through activating ER stress-responsive PERK-eIF2α-ATF4 signaling in HNSCC cells. Increased FGF19 is secreted and then recruited back to cells by its specific receptor FGFR4 and further activates Vimentin signaling, which in turn confines the role of MT in anti-metastasis

HNSCC remains a challenging clinical problem because of the persisting high rate of local and distant failure [39]. LNMets are the most important prognostic factor for patients with malignant tumors of epithelial origin, which portend worse survival of HNSCC patients [40]. However, the mechanisms by which malignant tumors evade the primary tumor site and metastasize to regional lymph nodes remain elusive. Here we report that high-dose MT not only induces ER stress-associated apoptosis in HNSCC cells but also promotes FGF19/FGFR4 signaling via activating ER stress-responsive PERK-eIF2α-ATF4 pathway (Fig. 7). Most importantly, upregulation of FGF19/FGFR4 axis confines the role of MT in suppressing metastasis and even triggers the switch of its function from anti-metastasis to metastasis promotion (Fig. 7).

In some cases, MT can directly stimulate mitochondrial differentiation and mitochondria-based apoptosis [41], while it appears that MT triggers mitochondria-based apoptosis in HNSCC cells under ER stress. Besides the appearance of c-Caspase 3 and c-Caspase 12, the expression levels of pro-apoptotic Bcl-2 family proteins, including Puma, Noxa and Bax, were also observed increase in MT-treated HNSCC cells. ATF4 regulates the cellular response to ER stress and its involvement in the ER stress-mediated upregulation of pro-apoptotic Bcl-2 family proteins has been well studied [36, 42]. We have demonstrated that MT results in the upregulation of ATF4 following ER stress in HNSCC cells, thus, ATF4-dependent transactivation of pro-apoptotic Bcl-2 family members is implicated in MT-induced ER stress-associated apoptosis.

While serum FGF19 is considered as a potential novel biomarker for HCC [43], it appears that serum FGF19 may not help improve the prognosis in HNSCC patients as there is no statistical difference in serum FGF19 between patients and healthy controls. In our prospective study of patients with HNSCC, we found higher levels of serum MT associated with increased FGF19 levels in tumor tissues, but these patients did not show a good correlation between the concentrations of serum MT and FGF19, suggesting MT is most likely involved in FGF19 upregulation in tumor cells. These findings may only reflect the cohort of HNSCC patients that we enrolled, and there is a possibility that many other factors related to MT levels in patients’ blood have not been fully considered. However, we at least provide a principle of concept that the determination of serum MT levels may enable to predict the levels of FGF19 in head and neck tumor tissues, which would be beneficial for FGFR4-based therapeutics.

HN30 and HN31 are the two cell lines respectively derived from primary lesions in the base of pharynx and lymph node metastatic lesions belonging to the same HNSCC patient [44]. Our previous study provides evidence that the highly invasive HN31 cells express higher levels of FGF19 than do the less invasive HN30 cells [25], suggesting the possible correlation between FGF19 expression levels and invasiveness of HNSCC cells. In this study, the observation that MT-LE HN30 cells progress to highly invasive cancer cells with elevated FGF19 expression prompted us to determine the specific role of FGF19 in cell aggressiveness associated with MT treatment. We found significantly increased FGF19 expression levels in MT-LE HN30 cells and depleting it in these cells suppressed cell motility. Crucially, loss of FGF19 attenuated metastatic potential of tongue tumors that were derived from MT-LE HN30 cells. These findings suggest that MT-induced increase of FGF19 gene expression shapes the role of MT in metastasis.

Intriguingly, the present study reveals that only high doses of MT can effectively activate ER stress-associated ATF4-dependent FGF19 upregulation in HNSCC cells, which redirects cell motility promotion to FGFR4-Vimentin signaling. This may partially explain why high-dose MT only has a limited effect on suppressing cell migration. It is noteworthy that epithelial HNSCC cells gain the capability to drive metastatic progression in an FGF19-dependent manner and develop malignancy when they are long exposed to high-dose MT. This data implicates that treatment with high-dose MT may change the epithelial plasticity and enable epithelial carcinoma cells to metastasis via FGF19 signaling. We previously uncovered that FGFR4 promotes EMT in HCC cells by activating GSK3β/β-catenin signaling [6]. The human Vimentin promoter was found to be upregulated by β-catenin/TCF-4 pathway or other mechanisms [45]. Nevertheless, considering the regulatory role of β-catenin may vary with the cellular background, in HNSCC cells, it remains undiscovered whether β-catenin is required for the regulation of Vimentin by FGFR4. Further exploration of the underlying mechanism is warranted, especially under the influence of high-dose MT. Besides the link between FGF19 expression and MT efficacy, the new knowledge regarding the involvement of FGF19 in the process of LNMets should enable improvements in prognostic and possibly therapeutic approaches to the management of malignant HNSCC.

As targeting FGFR4 becomes increasingly more feasible than directly targeting FGF19, highly selective FGFR4 inhibitors have become commercially available for preclinical tests [46–49], providing new approaches to treat a subgroup of FGF19-driven cancers. H3B-6527 has shown FGFR4 pathway modulation in Hep3B cells through the direct covalent binding to the ATP pocket of FGFR4 [47]. Although the pharmacodynamics and therapeutic efficacy of H3B-6527 have been evaluated in different mice models bearing HCC [47], none has shed light on other types of cancer. In the present study, we did not observe the potency of H3B-6527 in preventing the development of HN30 cell-derived tumors in mice, which may be due to the native low FGF19 expression in HN30 cells. However, tumor growth and aggressiveness in these tumors derived from MT-LE HN30 cells can be effectively suppressed by H3B-6527. This was attribute to the blockade of hyperactivated FGF19/FGFR4 signaling in MT-LE cells. Our data potentially indicates that FGF19 elevation is a strong predictor of H3B-6527 response in HNSCC cells.

In a xenograft model of HCC, H3B-6527 was administered orally either on a once-daily or twice-daily schedule [47]. We applied H3B-6527 at a high dose of 300 mg/kg once daily to orthotopic xenograft mice, but oral administration did not lead to tumor remission before tumor-bearing mice reached a moribund state. Therefore, to overcome this challenge, we switched to intratumoral treatment. In principle, intratumoral injections can be considered for any tumor where the primary lesion or its metastases are accessible [50], though there is only limited clinical use of non-systemic intratumoral therapy for cancers. In the present study, we demonstrate that direct injection into the tongue tumor not only reduces systemic exposure to avoid off-target toxicities but also decreases the amounts of H3B-6527 with stronger antitumor activity, which may provide a preclinical foundation for future opportunities for safer, more effective, and clinically practical non-systemic therapy.

## Conclusions

To our knowledge, this study is the first to interrogate the multifaceted role of MT in metastasis. Our findings provide novel mechanistic insight into tumor aggressiveness that is associated with MT-mediated upregulation of FGF19/FGFR4 signaling axis, suggesting that inactivating this molecular node may help actualize the promise of MT supplements in cancers.

## Supplementary Information

**Additional file 1: Table S1**. The clinicopathologic characteristics of sample sets. **Figure S1**. MT upregulates pro-apoptotic Bcl-2 family proteins in HNSCC cells. **Figure S2**. MT has no significant influence on systemic levels of FGF19 in HNSCC patients. **Figure S3**. FGF19 overexpression increases cell motility in HN30 cells. **Figure S4**. HN30 cells resistant to apoptosis after long exposure to MT.

## Data Availability

All data generated or analyzed during this study are included in this published article [and its supplementary information files].

## Abbreviations

3D: Three-dimensional
ATF4: Activating transcription factor 4
CLD: Chronic liver disease
ChIP-qPCR: Chromatin immunoprecipitation qPCR
DMEM: Dulbecco’s modified Eagle’s medium
eIF2α: Eukaryotic initiation factor 2 alpha
EMT: Epithelial-mesenchymal transition
ER: Endoplasmic reticulum
FGFs: Fibroblast growth factors
HCC: Hepatocellular carcinoma
H&E: Hematoxylin-and-eosin
HNSCC: Head and neck squamous cell carcinoma
IACUC: Institutional Animal Care and Use Committee
IHC: Immunohistochemstry
KO: Knockout
LNMets: Lymph node metastases
MT: Melatonin
MT-LE: Cells with melatonin long-term exposure
MT-SE: Cells with melatonin short-term exposure
qRT-PCR: Quantitative RT-PCR
ROS: Reactive oxygen species
SDs: Standard deviations
sgRNAs: Single-guide RNAs
shRNA: Short hairpin RNA
siRNA: Small interfering RNA
