# Supplementary material for: FGF19/FGFR4 signaling axis confines and switches the role of melatonin in head and neck cancer metastasis
Source: J Exp Clin Cancer Res. 2021 Mar 10;40:93. doi: 10.1186/s13046-021-01888-9 (PMC7945659; doi:10.1186/s13046-021-01888-9)
Supplement: Supplementary file 1 — Additional file 1: Table S1. The clinicopathologic characteristics of sample sets. Figure S1. MT upregulates pro-apoptotic Bcl-2 family proteins in HNSCC cells. Figure S2. MT has no significant influence on systemic levels of FGF19 in HNSCC patients. Figure S3. FGF19 overexpression increases cell motility in HN30 cells. Figure S4. HN30 cells resistant to apoptosis after long exposure to MT. [file 13046_2021_1888_MOESM1_ESM.rtf]

Supplementary Materials 

FGF19/FGFR4 signaling axis confines the role of melatonin in head and neck cancer metastasis
Liwei Lang, Yuanping Xiong, Nestor Prieto-Dominguez, Reid Loveless, Caleb Jensen, Chloe Shay, Yong Teng*


Correspondence to: yteng@augusta.edu


This PDF file includes: 
Supplementary Data Table S1
Supplementary Data Figure S1
Supplementary Data Figure S2
Supplementary Data Figure S3
Supplementary Data Figure S4


 
 


Table S1. The clinicopathologic characteristics of sample sets
	   Total	  MTlow (31)	 MThigh (25)	
Age, y				
Mean	61.3±7.54	60.24±7.45	62.16±7.49	
Tumor Sites				
Larynx	31	22	9	
Pharynx	25	9	16	
Histologic grade				
G1	14	13	1	
G2	28	12	16	
G3	14	6	8	
Lymph node metastasis				
Negative	29	24	5	
Positive	27	7	20	
Clinical Stages				
Ⅰ+Ⅱ	24	19	5	
Ⅲ+Ⅳ	32	12	20	


Fig. S1. MT upregulates pro-apoptotic Bcl-2 family proteins in HNSCC cells. 


Fig. S2. MT has no significant influence on systemic levels of FGF19 in HNSCC patients. (A) The secretion levels of FGF19 and MT in the blood samples from HNSCC patients and age-matched healthy controls measured by ELISA. (B) Spearman's rank correlation coefficient analysis of the correlation between the serum concentrations of MT and FGF19 in HNSCC patients.
                


Fig. S3. FGF19 overexpression increases cell motility in HN30 cells. (A) Increased Vimentin levels in FGF19 overexpressing (FGF19 O/E) HN30 cells compared with its parental cells and cells overexpressing empty vector (EV). (B) Increased cell migration in FGF19 O/E HN30 cells compared with its parental cells and cells overexpressing EV. Representative images and quantitative data from three independent experiments are shown in the left and right panels, respectively. **p<0.01.


Fig. S4. HN30 cells resistant to apoptosis after long exposure to MT. MT-SE and MT-LE HN30 cells were collected for Western blotting using antibodies against c-CASPASE 12 and c-CASPASE 3.
